# Within- and Between-Home Variability in Indoor-Air Insecticide Levels during Pregnancy among an Inner-City Cohort from New York City

**DOI:** 10.1289/ehp.9546

**Published:** 2006-12-11

**Authors:** Robin M. Whyatt, Robin Garfinkel, Lori A. Hoepner, Darrell Holmes, Mejico Borjas, Megan K. Williams, Andria Reyes, Virginia Rauh, Frederica P. Perera, David E. Camann

**Affiliations:** 1 Columbia Center for Children’s Environmental Health, Mailman School of Public Health, Columbia University, New York, New York, USA; 2 Southwest Research Institute, San Antonio, Texas, USA

**Keywords:** indoor air, insecticides, minority, pregnancy, residential

## Abstract

**Background:**

Residential insecticide use is widespread in the United States, but few data are available on the persistence and variability in levels in the indoor environment.

**Objective:**

The study aim was to assess within- and between-home variability in indoor-air insecticides over the final 2 months of pregnancy among a cohort of African-American and Dominican women from New York City.

**Methods:**

Women not employed outside the home were enrolled between February 2001 and May 2004 (*n* = 102); 9 insecticides and an adjuvant were measured in 48-hr personal air samples and 2-week integrated indoor air samples collected sequentially for 7.0 ± 2.3 weeks (*n* = 337 air samples).

**Results:**

Sixty-one percent of the women reported using pest control during the air samplings. Chlorpyrifos, diazinon, and propoxur were detected in 99–100% of personal and indoor samples (range, 0.4–641 ng/m^3^). Piperonyl butoxide (a pyrethroid adjuvant) was detected in 45.5–68.5% (0.2–608 ng/m^3^). There was little within-home variability and no significant difference in air concentrations within homes over time (*p* ≥ 0.2); between-home variability accounted for 88% of the variance in the indoor air levels of propoxur, 92% in chlorpyrifos, 94% in diazinon, and 62% in piperonyl butoxide (*p* < 0.001). Indoor and maternal personal air insecticide levels were highly correlated (*r* = 0.7–0.9, *p* < 0.001). Diazinon and chlorpyrifos levels declined 5-fold between 2001 and 2004 but were detected in all homes 1.5 and 2.5 years, respectively, after the U.S. Environmental Protection Agency ban on their residential use.

**Conclusion:**

Results showed that the insecticides were persistent in the home with little variability in air concentrations over the 2 months and contributed to chronic maternal inhalation exposures during pregnancy.

Residential pesticide use is widespread in the United States. Eighty-five percent of American households store at least one pesticide in their home, and approximately 10% of the conventional pesticides used annually in the United States are applied in and around the home (Adgate et al. 2000; Kiely et al. 2004). Many current-use insecticides are semivolatile (Lewis 2001) and are readily detected in indoor air after residential use. Postapplication air levels can be substantial, and inhalation has been shown to be a primary route of residential exposure in some but not all studies (Clayton et al. 2003; Pang et al. 2002; Whitmore et al. 1994; Whyatt et al. 2002, 2003). Although currently used insecticides tend to degrade rapidly in the outdoor environment because of the availability of water and ultraviolet light, which lead to hydrolytic and photolytic degradation, half-lives appear to be longer indoors (Lewis 2005). However, few data are available on the persistence of insecticides in the indoor environment over extended periods. The current study extends the research being conducted by the Columbia Center for Children’s Environmental Health (CCCEH) among inner-city mothers and newborns from New York City (Whyatt et al. 2003, 2004, 2005). Prior findings have shown widespread pesticide use among the CCCEH cohort: 87% of the 620 mothers report using some form of pest control during pregnancy, and 46% report using higher-toxicity methods (can sprays, pest bombs, and sprays by exterminators). Further, the organophophate insecticides chlorpyrifos and diazinon and the carbamate propoxur were detected in 99–100% of personal air samples collected from the mothers over 48 hr during pregnancy and in 29–55% of the blood samples collected from the mothers and newborns at delivery. Infants with the highest umbilical cord chlorpyrifos levels had significantly lower weight and length at birth (Whyatt et al. 2004) and significantly poorer mental and motor development at 3 years of age (Rauh et al. 2006). We undertook the current study to evaluate the extent of variability in indoor-air insecticide levels over the final 2 months of pregnancy and to assess whether indoor air concentrations contributed to chronic maternal prenatal inhalation exposures. We decided to monitor the third trimester because it corresponds to the spurt in brain growth during fetal development and may be a window of vulnerability (Eriksson 1997; Slotkin 1999). The study was also undertaken to evaluate the persistence of chlorpyrifos and diazinon in the indoor environment after the U.S. Environmental Protection Agency (EPA) 2001–2002 ban on their residential use (U.S. EPA 2000, 2001).

## Methods

The current study is part of a biomarker validation study and involved 102 women from the CCCEH cohort. Enrollment criteria for the CCCEH cohort have been described elsewhere (Perera et al. 2003; Whyatt et al. 2002, 2003). Women were enrolled through the prenatal clinics at New York Presbyterian and Harlem Hospital Centers in New York City between 2001 and 2004. To be eligible, women must have resided in northern Manhattan (north of 110th Street) or the South Bronx (south of Fordham Road) for at least 1 year before pregnancy and not be planning to move from the community before delivery. The women must also have been registered to deliver at New York Presbyterian and Harlem Hospital Center. In addition to the enrollment criteria for the full CCCEH cohort, enrollment into the current study was restricted to women who were not employed outside the home at the time of enrollment so as not to confound pesticide exposures in the home with pesticide exposures in the workplace. Workplace exposures would be difficult for the woman to quantify with any reliability. Study procedures, including personal and indoor air monitoring as well as the collection of biologic samples, were explained at enrollment, and the date for the indoor air monitoring was scheduled to commence around the 32nd week of pregnancy. Results of the biologic samples will be published separately. A signed, institutional review board–approved consent form was obtained from each study participant at enrollment.

Demographics for women in the current study (*n* = 102) are reported in Table 1. They are comparable to those of women in the remaining CCCEH cohort (*n* = 612) in terms of maternal age at enrollment (*p* = 0.8, group *t*-test), ethnicity (chi-square = 1.2, *p* = 0.6), marital status (chi-square = 0.7, *p* = 0.4), and annual household income (chi-square = 0.2, *p* = 0.6). In addition, a similar proportion of women in the current study, compared with those in the remaining CCCEH cohort, reported that pest control methods were used during pregnancy (85.7 vs. 86.9%, chi-square = 0.1, *p* = 0.7). However, a greater proportion of women in the current study than those in the remaining CCCEH cohort reported having less than a high school education (46 vs. 34%, chi-square = 5.8, *p* = 0.02) and a greater proportion of the women in the remaining CCCEH cohort than those in the current study reported that they were employed at some point during pregnancy (55.7 vs. 41%, chi-square = 7.4, *p* < 0.01). Most of the women (96%) in the cohort lived in residential apartment buildings; the remaining women lived in multifamily housing (*n* = 3) or in combined residential/commercial buildings (*n* = 1).

### Questionnaire data

A full questionnaire was administered to the woman at the first home visit during the 32nd week of pregnancy. It included basic demographic information, neighborhood and home characteristics, history of active and passive smoking, and history of exposures, including pesticide use during pregnancy. In addition, at each bi-weekly home visit, a short 10- to 15-min questionnaire was administered that gathered exposure information specific to pesticide exposures during the preceding 2 weeks. Questions included whether pests (specifically cockroaches, mice, rats, or other pests) were seen in the home and, if so, for each pest type, how frequently they were seen over the 2 weeks (less than once per week, 1–3 times per week, 4–6 times per week, or daily) and whether any pest control measures were used over the prior 2 weeks either by an exterminator or by others in the home (the participant, building superintendant, or other household member). If pest control measures were used, information was gathered on which methods were used, who used them, what pest they were used for, and how frequently they were used. Because 47% of the cohort was Spanish speaking, the interviews were conducted in both Spanish and English by a fully bilingual research staff. A total of 333 2-week questionnaires were gathered from the study participants; 74% of women completed three or more questionnaires.

### Indoor and personal air sampling

The indoor air monitoring was conducted continuously beginning during the 32nd week of pregnancy until delivery. Two-week integrated indoor air samples were collected every 2 weeks throughout the monitoring period. We monitored the air using a pump with a 0.5-L/min flow rate attached to a polyurethane foam (PUF) sampler that had a 2.5-μm inlet cut fitted with a 25-mm quartz fiber filter and a foam cartridge backup to capture semivolatile vapors and aerosols, as previously described (Whyatt et al. 2002, 2003). The pump was attached to a battery that operated the pump at 0.5 L/min continuously throughout the 2 weeks. Every other week, the PUF sampler was removed and a new sampler and battery attached. The indoor air monitoring continued until the woman went into labor, and was conducted for an average of 7.0 ± 2.3 (range, 1.9–12.4) weeks per home, with an average of 3.5 ± 1.1 (range, 1–6) 2-week integrated indoor air samples collected per home. The monitorings continued for ≥ 6 weeks in 80% of the homes. In four cases, an additional 2-week integrated indoor air sample was collected after delivery, because predelivery samples had been lost through a laboratory error. The personal air sampling was collected from the mother over 48 hr during the 32nd week of pregnancy. Women in the cohort were asked to wear a small backpack containing a personal ambient air monitor during the daytime hours for 2 consecutive days and to place the monitor near the bed at night. The personal air sampling pump operated continuously at 4 L/min over this period, collecting particles of ≤ 2.5 μm in diameter on a quartz microfiber filter and collecting semivolatile vapors and aerosols on a PUF cartridge backup. A 48-hr personal air sample was collected from 96 of 102 (94%) women in the study. The indoor sampling was initiated at the conclusion of the 48-hr personal air sampling.

Processing and analysis of indoor and personal air samples was undertaken as described previously (Whyatt et al. 2002, 2003). Briefly, immediately after collection, the indoor and personal air monitoring samples were brought to the molecular epidemiologic laboratory at the Mailman School of Public Health, inventoried, and frozen. Once per month, air samples were shipped on dry ice to Southwest Research Institute and stored at −12° C. Within 10 days of arrival, the PUF plug and filter were placed in a Soxhlet extractor (Corning Inc., Corning, NY), spiked with terphenyl-d_14_ as a recovery surrogate and extracted with 6% diethyl ether in hexanes for 16 hours. The extract was then concentrated to 1 mL and frozen at −12° C before analysis. Pesticides were analyzed by gas chromatography/mass spectrography as described previously (Whyatt et al. 2002, 2003). Nine insecticides were measured in the indoor and personal air samples: the organophosphates chlorpryifos, diazinon, malathion, and methyl parathion; the carbamates propoxur, bendiocarb, and carbofuran; and the pyrethorids *cis*-and *trans*-permethrin. In additional, piperonyl butoxide, an adjuvant that is frequently added to pyrethroid formulations, was measured as an indicator of pyrethroid use.

### Statistical analyses

Descriptive analyses preceded formal hypothesis testing. We calculated the percentage of indoor and personal air samples with pesticide levels above the limit of detection (LOD) as well as the range of pesticide concentrations in the samples. We calculated arithmetic means ± SDs for the pesticides detected in > 45% of samples. Before statistical analyses, pesticide values were log-transformed to normalize their distributions. Values below the LOD were assigned a value of 0.5 × LOD. We used Pearson correlation coefficients or Spearman’s rank-order (for pesticides detected in < 45% of samples) to examine correlations between pesticide levels in indoor air and personal air samples. We used analysis of variance (ANOVA) to test whether pesticides levels varied significantly by season and year of monitoring (2001–2004) or among the following groups: women not using any pest control methods; women using nonspray methods only (sticky traps, bait traps, boric acid, and gels); and women using higher-toxicity pesticides (can sprays, pest bombs, or sprays by an exterminator, with or without nonspray methods). If levels differed significantly among the groups, we used the least significant difference test to determine which groups varied significantly. We used *t*-tests to test whether pesticide levels differed significantly by ethnicity, and chi-square analyses to test whether the proportion of women using pest control measures over the final 2 months of pregnancy was related to pest sightings in the home. A mixed-effects model was used to determine whether the within-home or the between-home variability in the repeat indoor air samples contributed more to overall differences in measured pesticide levels, after controlling for season and year of delivery. Except for the Spearman’s rank and chi-square analyses, all statistical testing was conducted on the log-transformed data. Results were considered significant at *p* < 0.05.

## Results

Table 2 shows the number of women reporting that pests were sighted in the home or that pest control measures were used over the 7.0 ± 2.3 weeks of the indoor air sampling. Ninety (91%) of the women reported that pests were sighted in the home. Cockroaches were the pests sighted most frequently, with 80% of the women reporting that cockroaches were sighted at some point during the study period and 48% reporting that cockroaches were sighted at least once every 2 weeks. Over half the women also reported sighting mice in the home, with 22% reporting that mice were seen at least once every 2 weeks during the study period. Sighting of rats and other pests was less common. Sixty women (61%) reported using some form of pest control during the 7.0 ± 2.3 weeks: 32% reported using lower-toxicity methods only, and 29% reported using one or more of the higher-toxicity methods (can sprays, sprays by exterminator, and pest bombs) with or without the lower-toxicity methods. The lower-toxicity methods were applied by the women themselves in 32% of cases, by others in the household in 52%, and by an exterminator in 16%. Can sprays and pest bombs were applied by the women themselves in 41% of the cases and by others in the household in 59%. Sprays by an exterminator were reported by 18% of the women using pest control. In addition, 3 of 99 (3%) women reported use of illegal street pesticides (Tempo or Tres Pasitos). Consistent with our prior findings (Whyatt et al. 2002, 2003, 2005), most of the pesticide use was targeted at cockroach control. However, several pest control methods (sticky traps, bait traps, and illegal pesticides) were applied to control both cockroaches and rodents. None of the methods were targeted at pests other than cockroaches and rodents. There was no significant difference in the proportion of women using any form of pest control or in the proportion using the higher- and lower-toxicity pest control methods, by year of enrollment (2001 through 2004, chi-square = 2.1, *p* = 0.9). Nor was there any significant difference in pesticide use habits between African Americans and Dominicans (chi-square = 1.4, *p* = 0.5). Women who reported that pests were sighted in the home were significantly more likely to report use of pest control (chi-square = 6.1, *p* = 0.01).

Results on pesticide levels in the indoor and personal air samples are presented in Tables 3 and 4. Of the nine compounds measured, chlorpyrifos, diazinon, and propoxur were detected in 99.7–100% of the maternal personal and indoor air samples, and piperonyl butoxide (an adjuvant to pyrethorid insecticides) was detected in 45.5 and 68.5% of indoor and personal air samples, respectively. The remaining insecticides were detected less frequently (0–17% of samples). As can be seen from comparing Tables 3 and 4, the average levels of these four compounds in the maternal and indoor air samples were similar. Further, as Table 5 shows, the correlations between the levels of chlopryrifos, diazinon, and propoxur in maternal personal and indoor air samples were highly significant (Pearson *r* = 0.70–0.90, *p* < 0.001, Pearson correlation coefficient). A somewhat weaker correlation was seen between maternal personal and indoor air levels of piperonyl butoxide (*r* = 0.56–0.72; Table 5) but the association was again highly significant (*p* < 0.001).

**Figure 1:**
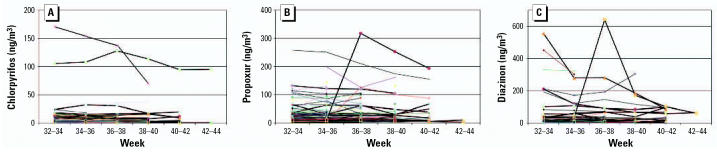
Two-week integrated indoor air insecticide levels (ng/m^3^) between the 32nd week of pregnancy and delivery (*n* = 102 homes). (*A*) Chlorpyrifos. (*B*) Propoxur. (*C*) Diazinon.

Figure 1 shows the indoor air levels of chlorpyrifos, diazinon, and propoxur over the 7.0 ± 2.3 weeks of sequential indoor air sampling. There was relatively little within-home variability in most of the homes in indoor air concentrations of these insecticides. For this reason, the correlations among concentrations detected in each 2-week period for all three insecticides were highly significant (*r* = 0.75–0.96, *p* < 0.001, Pearson correlation coefficient). Nor was there any significant difference in within-home air levels of these three insecticides in the mixed-effects model (all *p*-values > 0.3, controlling for season and year of the monitoring). Between-home variability accounted for 92% of the variance in chlorpyrifos levels, 94% in diazinon, and 87.7% in propoxur (*p* < 0.001).

Indoor air levels of piperonyl butoxide were somewhat more variable than for the insecticides, but the correlations among concentrations detected in each 2-week period were highly significant (*r* = 0.57–0.73, *p* < 0.001, Pearson correlation coefficient). Further, there was no significant within-home difference in indoor air over the 6–10 weeks of sequential sampling (*p* > 0.2, mixed-effects model). By contrast, between-home variability explained 62.1% of the variance in piperonyl butoxide levels (*p* < 0.001, mixed-effects model).

Indoor air levels of piperonyl butoxide were significantly correlated with air levels of both *cis*- and *trans*-permethrin during each 2-week sampling period (*r* = 0.42–0.71, *p* ≤ 0.001, Spearman’s rank) and also when averaged over the 6–8 weeks of sequential sampling (*r* = 0.56, *p* < 0.001, *n* = 91 for *cis*-permethirn and *r* = 0.50, *p* < 0.001, *n* = 91, for *trans*-permethrin, Spearman’s rank; data not shown). Piperonyl butoxide levels were generally < 90 ng/m^3^. However, there was one high excursion (> 600 ng/m^3^) during one of the 2-week monitoring periods. Interestingly, during the same 2-week period, this home showed the highest *cis*-permethrin (54 ng/m^3^) and *trans*-permethrin (102 ng/m^3^) levels of the homes sampled. The woman herself reported the use of can sprays and pest bombs during the 2 weeks.

**Figure 2:**
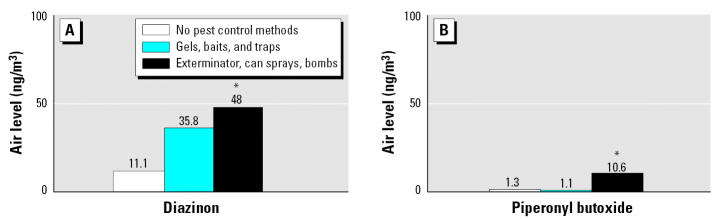
Average indoor air levels (ng/m^3^) of diazinon (*A*) and piperonyl butoxide (*B*) by maternal self-reported pesticide use between the 32nd week of pregnancy and delivery (*n* = 102 homes). *p < 0.05, ANOVA.

Figure 2 shows average indoor air levels of diazinon and piperonyl butoxide by the women’s self-report of pesticide usage over the 7.0 ± 2.3 weeks of sequential indoor air sampling. Chlorpryfos air levels were not associated with self-reported pesticide use (data not shown). Indoor air levels of propoxur were somewhat higher in homes where the woman reported using can sprays, sprays by exterminator, and/or pest bombs. However, the difference was of borderline significance only among homes monitored during one of the 2 weeks (*p* = 0.06, ANOVA; data not shown) Average indoor air levels of diazinon and piperonyl butoxide were significantly associated with maternal self-reported pesticide use (*p* < 0.05, ANOVA; Figure 2). Specifically, diazinon levels were significantly higher among women who reported using one or more of the higher-toxicity methods (can sprays, sprays by an exterminator, and/or pest bombs) during the indoor air sampling compared with women who reported using no pest control (*p* = 0.02, ANOVA). Piperonyl butoxide air levels were significantly higher among women reporting using the higher-toxicity methods compared with women who reported using no pest control (*p* = 0.02, ANOVA) as well as with women who reported using the lower-toxicity methods only (traps, baits, and gels; *p* = 0.009, ANOVA). There was no significant difference in either maternal personal air levels or average indoor air levels between African Americans and Dominicans (data not shown).

Indoor air levels of chlorpyrifos and diazinon decreased significantly between homes monitored in 2001 compared with homes monitored in 2004. For chlorpyrifos, air levels decreased 5-fold from an average of 12.4 ± 29.0 ng/m^3^ among homes monitored in 2001 (*n* = 32) to an average of 2.4 ± 1.9 ng/m^3^ among homes monitored in 2004 (*n* = 16) (*p* = 0.018, ANOVA). The linear trend test was also significant (*p* = 0.005). For diazinon, air levels decreased almost 5-fold over the same period from an average of 47.4 ± 67.3 ng/m^3^ among homes monitored in 2001 (*n* = 32) to an average of 10.0 ± 11.8 ng/m^3^ among homes monitored in 2004 (*n* = 16) (*p* = 0.001, ANOVA). The linear trend test was again significant (*p* < 0.01). Consistent with the results in indoor air levels and with our prior publications (Whyatt et al. 2003, 2004), a decrease in maternal personal air levels from 2001 to 2004 was seen for both chlorpryifos (from 9.3 ± 17.3 to 2.2 ± 2.0 ng/m^3^, *p* = 0.07, ANOVA) and diazinon (from 46.2 ± 68.7 to 9.5 ± 12.1 ng/m^3^, *p* = 0.001, ANOVA). However, both insecticides were still detected in virtually all of the maternal personal and indoor air samples 1.5–2.5 years after the U.S. EPA ban on their residential use of diazinon and chlorpyrifos, respectively (U.S. EPA 2000, 2001). There was no significant difference in either maternal personal or indoor air levels of propoxur or piperonyl butoxide by year of the monitoring.

Levels of the four compounds in the indoor air samples tended to be higher in monitorings initiated in the spring and/or summer (67% of homes) compared with those initiated in the winter and/or fall (33% of homes). The seasonal difference was significant for chlorpyrifos (*p* = 0.03, ANOVA) and for piperonyl butoxide (*p* = 0.01, ANOVA; data not shown). The women also reported seeing cockroaches somewhat more frequently in the spring and summer but the difference was not significant (chi-square = 1.3, *p* = 0.25). Information on additional factors that may have influenced seasonal variability (i.e., ventilation rates and use of air conditioning) was not gathered in the current study.

## Discussion

In the current study we measured nine insecticides and an adjuvant in 2-week integrated indoor air samples collected sequentially over the final 2 months of pregnancy among homes of African-American and Dominican women residing in New York City. Three insecticides (chlorpyrifos, diazinon, and propoxur) were detected in air samples from all homes. Piperonyl butoxide, a synergist added to pyrethorid formulations, was also frequently found. Our results are consistent with prior research indicating that semivolatile insecticides are readily detected in indoor air after residential use (Bradman and Whyatt 2005; Lewis 2005). Chlorpyrifos, diazinon, and propoxur have been among the most frequently detected insecticides in indoor air samples in the United States (Bradman et al. 2006; Clayton et al. 2003; Morgan et al. 2005; Pang et al. 2002; Whitmore et al. 1994). However, our results are surprising in that the indoor air levels of these insecticides in the 2-week integrated indoor air samples remained remarkably stable within most homes over the 6–8 weeks of sequential sampling. This is not the pattern that has been seen in most prior research. However, unlike our design, these prior investigations have measured insecticide air concentrations over shorter time frames to evaluate day-to-day fluctuations in concentrations. By contrast, our air sampling methodology was not designed to look at day-to-day fluctuations but rather at chronic exposure levels during the final 2 months of pregnancy.

The prior research indicates that air levels of semivolatile insecticides drop fairly rapidly immediately after applications, as the pesticides are absorbed into furnishings or dissipate to the outdoor air (Lewis 2005). Chlorpyrifos, for example, was shown to decline approximately 2-fold between air samples collected 1–2 days after application compared with air levels 2 weeks after application (Lewis et al. 1994, 2001). Diazinon levels were also shown to drop 2-fold or more from between day 1 and day 12 after applications. In a study that measured indoor air concentrations of chlorpyrifos sequentially over 2 weeks after application, levels were found to peak in a two-phase process: an aerosolized particle phase with concentration peaking on surface areas after 36 hr and a gas phase that began 12 hr after application and continued for at least 2 weeks, with levels peaking on surface areas at 72 hr and then declining (Gurunathan et al. 1998). A more recent study of 10 homes measured chlorpyrifos concentrations sequentially in indoor air and on plush toys for 2 weeks after applications (Hore et al. 2005). In most homes, air levels peaked by 2 days after application and then declined over the 2 weeks. However, chlorpyrifos concentrations on the plush toys continued to rise in all homes over the 2-week period, with levels averaging 3-fold to 4-fold higher by day 11 compared with day 1 after application (Hore et al. 2005). These results indicate that plush furnishing in the home can provide a sink for the insecticide and may account for the persistence in the indoor-air insecticide concentrations that we have documented in the current study. Prior research has also shown that air concentrations of semivolatile insecticides can still be 20–30% of those on the day of application after as many as 21 days, indicating that the compounds can be quite persistent in the indoor environment (Lewis 2005). In the present study, because of the stability of the insecticides in air in most homes, almost all (90%) of the variance in the indoor air levels was explained by between-home variability. This pattern was similar for diazinon and propoxur, which were still in use during the study period, and for chlorpyrifos, which had been banned for residential use before most of the homes in the current study were monitored.

Specifically, air samplings were conducted on more than half the homes before the December 2002 termination of all retail sales of diazinon for residential use (U.S. EPA 2001). The fact that diazinon concentrations in the indoor air samples were significantly associated with self-reported pesticide use also supports ongoing use of this insecticide, at least for part of the study. In addition, in a survey conducted of 135 stores in the study area during June–July 2002, we found products containing diazinon in approximately 40% of stores (Carlton et al. 2004). A follow-up 1 year later found diazinon products in 18% of stores (including 80% of supermarkets) (Carlton et al. 2004). Participants may also have used products containing diazinon and chlorpyrifos that were purchased before the ban and stored in the home. There was little within-home variability in diazinon levels seen in the current study, and between home variability accounted for 94% of the variance in air concentrations of this insecticide (with year of measurement controlled). However, our results also indicate that the ban has effectively reduced the indoor air concentrations over time: Diazinon levels were almost 5-fold on average lower among homes monitored in 2004 than those monitored in 2001, but was still detected in all homes including those sampled 1.5 years after the ban.

Propoxur is an insecticide licensed for use against a wide variety of indoor insects, including cockroaches (U.S. EPA 1997). Although indoor air concentrations of propoxur were not significantly associated with self-reported pesticide use in the current study, levels tended to be higher in homes using can sprays, sprays by exterminators, or pest bombs. Further, air concentrations of propoxur did not differ significantly between homes monitored during 2004 (average of 54.7 ng/m^3^) and homes monitored in 2001 (average of 38.3 ng/m^3^), indicating that use of this insecticide was ongoing during the study period. As with diazinon, there was no significant within-home variability in propoxur levels over the 6–8 weeks of sequential sampling, and between-home variability accounted for almost 90% of the variance in the insecticide levels.

Unlike diazinon and propoxur, chlorpyrifos use had been largely curtailed before the sampling of most homes in the current study. Specifically, 71% of the homes were sampled after the December 2001 ban on residential use (U.S. EPA 2000). Further, the ban appears to have been effective at eliminating chlorpyrifos sales in inner-city communities (Carlton et al. 2004). The fact that we did not see any association between indoor air levels of chlorpryifos and maternal self-reported pesticide use also supports the hypothesis that most of the chlorpyrifos concentrations detected in the indoor air samples resulted from prior use. Nonetheless, chlorpyrifos was detected in indoor air samples from all homes including those monitored 2.5 years after the ban, showing that it is quite persistent in the indoor environment. As with the other insecticides, there was little within-home variability in chlorpyrifos air concentrations over the 6–8 weeks of sequential sampling. However, air concentrations were 5-fold lower on average among homes monitored in 2004 than in 2001.

Several lines of evidence suggest that the pyrethoid insecticides are replacing the organophosphates chlorpyrifos and diazinon for residential use (Lewis 2005; Surgan et al. 2002). Most pyrethoids have low volatilities and are infrequently detected in indoor air samples (Lewis 2005). Therefore, we measured piperonyl butoxide as an indicator of pyrethroid use. Piperonyl butoxide is a synergist added to pyrethroid formulations to enhance the insecticidal activities by inhibiting the metabolitic degradation of the pyrethoids (Devine and Denholm 1998). It is both more volatile and more stable in indoor air and dust than pyrethroids (Fischer and Eikmann 1996). One study found that piperonyl butoxide persisted for at least 2 weeks after a single cockroach treatment on toys and in dust in a kindergarten (Fischer and Eikmann 1996). Piperonyl butoxide was detected in 45–68% of the indoor and personal air samples in the present study, whereas the pyrethroids *cis*-permethrin and *trans*-permethrin were detected considerably less frequently (in 14–17% of samples). However, indoor air levels of piperonyl butoxide were significantly correlated with those of *cis*- and *trans*-permethrin, suggesting that permethrin use accounted at least partly for the piperonyl butoxide levels seen in the present study. Indoor air levels of piperonyl butoxide were more variable than those of the three insecticides, but between-home variability still accounted for 61% of the variance in the piperonyl butoxide levels. There was no significant change in indoor air levels of piperonyl butoxide among homes monitored between 2001 and 2004, suggesting that, contrary to expectations, the pyrethroids may not be replacing the organophosphates among this cohort. This is surprising because the proportion of women reporting that can sprays, exterminator sprays, and pest bombs were used during pregnancy did not change between 2001 and 2004, but use of chlorpyrifos and diazinon appears to have been sharply reduced or eliminated. However, it is also possible that pyrethroid use may have already increased before the 2001 start date of the present study. Additional research is ongoing regarding replacement insecticides among women in the CCCEH cohort.

The present study confirms prior research results that indoor air levels of semivolatile insecticides contribute to personal inhalation exposures (Bradman and Whyatt 2005; Pang et al. 2002; Whitmore et al. 1994). In the present study, inhalation exposures of the mother were measured by personal air sampling over 48 hr during the 32nd week of pregnancy. A highly significant correlation was seen for all three insecticides (diazinon, propoxur, and chlorpyrifos) between maternal personal air levels and indoor air levels during each 2-week sampling period. Results indicate that the indoor air levels accounted for most (60–80%) of the variance in the maternal personal air levels. It is remarkable that the correlation between the insecticides in the maternal personal air sample and indoor air samples was as strong for the indoor samples collected 8 weeks after the maternal air sampling as it was for the indoor air sample collected immediately after the maternal personal air sample. This is again attributed to the fact that indoor air levels of these insecticides remained relatively constant within the home. Collectively, these results indicate that the women in the cohort received chronic inhalation exposures to the insecticides during pregnancy. The present study is part of a biomarker validation study, and enrollment was restricted to women who were not employed away from home at the time of the indoor air monitoring, so as not to confound pesticide exposures in the home with pesticide exposures in the workplace. Thus it is possible that residential exposures contribute less to personal exposures among women employed outside the home. However, prior research has also documented that within-home exposures are generally major contributors to personal exposures because of the greater time spent in the home relative to other environments (Sundel 2004).

Research is currently ongoing within the CCCEH cohort to evaluate whether there are any potential health effects associated with these exposures. Findings to date have shown that among the CCCEH infants born before the residential ban on chlorpyrifos, the amount of chlorpyrifos in the umbilical cord blood at birth was significantly inversely associated with the baby’s birth weight and length (Whyatt et al. 2004, 2005) and with the child’s mental and motor development at 3 years of age (Rauh et al., in press). In addition, children with the highest chlorpyrifos blood levels at birth were significantly more likely by 3 years of age to manifest symptoms of attentional disorder, attention deficit hyperactivity disorder, and pervasive personality disorder, based on parental report using the Child Behavior Checklist (Rauh et al. 2006). The cohort is currently being followed and analyses will be undertaken to determine whether there are any residual health effects associated with the low chlorpyrifos levels among infants born after the residential ban. Research is also ongoing to evaluate the relationship between insecticide levels in indoor air samples and those in biologic samples collected from the mother during pregnancy and from the mother and newborn at delivery.

## Figures and Tables

**Table 1:** Demographics of study women (*n* = 102).

Variable	Value [no. (%)]
Maternal age (mean ± SD)^a^	25.2 ± 4.9
Race/ethnicity^a^	
African American	31 (31)
Dominican	68 (68)
Other	1 (1)
Marital status^a^	
Never married	69 (69)
Married^b^	24 (24)
Separated, widowed, divorced	7 (7)
Education^a^	
< High school degree	46 (46)
High school diploma or GED	33 (33)
Some college (< 4 years)	20 (20)
College degree (4-year)	1 (1)
Household income (US$)^a^		
< 10,000	36 (42.9)
10,000–30,000	36 (42.9)
> 30,000	12 (14.3)

GED, general educational development.

a Missing values: maternal age (*n* = 3), ethnicity (*n* = 2), marital status (*n* = 2); education (*n* = 2); income (*n* = 18).

b Includes women living as married with same partner > 7 years.

**Table 2:** Number of women (%) reporting sighting of pests and use of pest control measures in the home between the 32nd week of pregnancy and delivery.^a^

Report	No. (%)
Total no. with pest sightings (*n* = 99)	90 (91)
Sightings of cockroaches (*n* = 99)	79 (80)
Sighting of rodents (mice or rats) (*n* = 99)	51 (52)
Sightings of other pests (*n* = 99)	21 (21)
Total no. using pest control measures (*n* = 99)	60 (61)
Used lower-toxicity methods only (*n* = 98)^b^	31 (32)
Used one or more higher-toxicity methods (*n* = 98)^c^	28 (29)

a Missing data: pest sightings (*n* = 3); specific pest control methods (*n* = 4).

b Sticky traps, gels, bait traps, boric acid.

c Can sprays, pest bombs, or sprays by an exterminator with or without the lower-toxicity methods.

**Table 3:** Air concentrations of 10 insecticides and a pesticide adjuvant in 2-week indicated air samples collected from 102 homes between the 32nd week of pregnancy and delivery.^a^

		Air concentrations (ng/m^3^)^a^		
Insecticide	No. > LOD/total samples (%)	Median	Mean ± SD^b^	Range
Organophosphates				
Chlorpyrifos	336/337 (99.7)^c^	3.0	6.9 ± 17.0	< 0.4–171
Diazinon	337/337 (100)^c^	7.4	28.5 ± 62.6	0.4–641
Methyl parathion	0/180 (0)^b^	< 0.2	NC	NC
Malathion	0/175 (0)^b^	< 0.2	NC	NC
Carbamates				
Propoxur	319/319 (100)^c^	26.1	37.8 ± 38.8	1.1–317
Bendiocarb	19/215 (8.8)^c^	< 0.2	NC	< 0.2–92.6
Carbofuran	0/245 (0)	< 0.2	NC	NC
Pyrethroids				
Piperonyl butoxide^d^	133/292 (45.5)^c^	< 0.2	3.9 ± 16.9	< 0.2–608
Permethrin				
*trans*-Permethrin	47/337 (13.6)^c^	< 0.1	NC	< 0.1–164
*cis*-Permethrin	57/337 (16.9)^c^	< 0.4	NC	< 0.4–125

NC, not calculated.

a In four homes, samples were additionally collected for 2 weeks after delivery because predelivery samples had been lost through a laboratory failure.

b Mean ± SD was calculated if the pesticide was detected in ≥ 45% of samples; levels in samples without detections were set at one-half of the detection limit.

c Air concentration could not be calculated for remaining sample(s) because of interference peaks or quality control issues.

d Not itself a pyrethroid, but it is an adjuvant and indicator of exposure to pyrethroids.

**Table 4:** Air concentrations of 10 insecticides and pesticide adjuvant in personal air samples collected over 48 hr from the mother during the 32nd week of pregnancy.

		Air concentrations (ng/m^3^)^a^		
Insecticide	No. > LOD/total samples (%)	Median	Mean ± SD^b^	Range
Organophosphates				
Chlorpyrifos	95/96 (99.0)^b^	2.8	6.2 ± 11.1	< 0.4–83.4
Diazinon	96/96 (100)^b^	8.6	27.2 ± 60.4	1.0–427.5
Methyl parathion	0/54 (0)^b^	< 0.2	NC	NC
Malathion	1/57 (1.8)^b^	< 0.2	NC	< 0.2–16.1
Carbamates				
Propoxur	96/96 (100)^b^	18.7	31.9 ± 45.6	1.7–342.9
Bendiocarb	10/68 (14.7)^b^	< 0.2	NC	< 0.2–27.5
Carbofuran	0/65 (0)	< 0.2	NC	NC
Pyrethroids				
Piperonyl butoxide^c^	61/89 (68.5)^b^	0.4	3.1 ± 11.4	< 0.2–98.2
Permethrin				
*trans*-Permethrin	14/96 (14.5)^b^	< 0.1	NC	< 0.1–7.5
*cis*-Permethrin	12/96 (12.5)^b^	< 0.4	NC	< 0.4–9.4

NC, not calculated.

a Mean ± SD was calculated if the pesticide was detected in ≥ 45% of samples; levels in samples without detections were set at one-half of the detection limit.

b Air concentration could not be calculated for remaining sample(s) because of interference peaks or quality control issues.

c Not itself a pyrethroid, but it is an adjuvant and indicator of exposure to pyrethroids.

**Table 5:** Correlation^a^ between levels of insecticides and a pesticide adjuvant in maternal personal air compared with those in 2-week integrated indoor air samples collected between the 32nd week of pregnancy and delivery.

	1st 2 weeks^b^ (*n* = 91)	2nd 2 weeks^b^ (*n* = 85)	3rd 2 weeks^b^ (*n* = 72)	4th 2 weeks^b^ (*n* = 49)	Average^c^ (*n* = 96)
Chlorpyrifos	0.86	0.82	0.85	0.87	0.85
Diazinon	0.89	0.87	0.88	0.88	0.90
Propoxur	0.72	0.70	0.75	0.70	0.76
Piperonyl butoxide	0.72	0.60	0.68	0.56	0.59

a All *p*-values < 0.001, Pearson’s correlation coefficient.

b Of indoor air samplings. Missing: propoxur: 1st 2 weeks (*n* = 5); 2nd 2 weeks (*n* = 4); 3rd 2 weeks (*n* = 4); 4th 2 weeks (*n* = 3); piperonyl butoxide: 1st 2 weeks (*n* = 11); 2nd 2 weeks (*n* = 11); 3rd 2 weeks (*n* = 10); 4th 2 weeks (*n* = 8).

c Average levels of all indoor air samples collected in each home between the 32nd week of pregnancy and delivery.
